# Intrinsic properties of germinal center‐derived B cells promote their enhanced class switching to IgE

**DOI:** 10.1111/all.12679

**Published:** 2015-07-24

**Authors:** F. Ramadani, N. Upton, P. Hobson, Y.‐C. Chan, D. Mzinza, H. Bowen, C. Kerridge, B. J. Sutton, D. J. Fear, H. J. Gould

**Affiliations:** ^1^ Randall Division of Cell and Molecular BiohphysicsKing's College LondonLondonUK; ^2^ Division of Asthma, Allergy and Lung BiologyKing's College LondonLondonUK; ^3^ Medical Research Council and Asthma UK CentreAllergic Mechanisms in AsthmaLondonUK

**Keywords:** allergy, germinal center, human B cells, IgE class switching

## Abstract

**Background:**

Research on the origins and development of human IgE‐expressing (IgE^+^) cells is required for understanding the pathogenesis of allergy and asthma. These studies have been thwarted by the rarity of IgE^+^ cells *in vivo* and the low frequency of class switch recombination (CSR) to IgE *ex vivo*. To determine the main source of IgE^+^ cells, we investigated the relation between the phenotypic composition of tonsil B cells and the CSR to IgE *ex vivo*.

**Methods:**

Human tonsil B cells were analyzed by flow cytometry (FACS) and cultured with IL‐4 and anti‐CD40 to induce CSR to IgE. Naïve, germinal center (GC), early GC (eGC), and memory tonsil B cells were isolated by FACS, and their capacities for IL‐4 and anti‐CD40 signaling, cell proliferation, and *de novo* class switching to IgE were analyzed by RT‐PCR and FACS.

**Results:**

B cells from different tonsils exhibited varying capacities for CSR to IgE *ex vivo*. This was correlated with the percentage of eGC B cells in the tonsil at the outset of the culture. Despite relatively poor cell viability, eGC and GC B‐cell cultures produced the highest yields of IgE^+^ cells compared to naïve and memory B‐cell cultures. The main factors accounting for this result were the strength of IL‐4R and CD40 signaling and relative rates of cell proliferation.

**Conclusions:**

This study shows that the maturation state of tonsil B cells determines their capacity to undergo class switching to IgE *ex vivo*, with the GC‐derived B cells yielding the highest percentage of IgE^+^ cells.

IgE provides immune defense against multicellular parasites and venoms but also mediates adverse reactions to otherwise harmless environmental antigens (allergens) in allergic disease and asthma 1, 2. The increasing prevalence of IgE‐mediated allergic disease is alarming. Yet very little is known about the development of IgE^+^ cells 3 due to the rarity of these cells in circulation and inaccessibility in tissues 4.

Mature B cells develop from IgM^+^ B‐cell precursors, generated in the bone marrow, and must undergo class switch recombination (CSR) to express the ‘switched’ isotypes (IgG, IgA, and IgE) 5, 6. CSR from IgM to another antibody class requires recombination of the DNA sequence encoding the expressed Ig heavy‐chain variable region linked to the μ heavy‐chain constant region (CH) with the CH of another antibody class in the tandem array of CH germline genes in the Ig heavy‐chain locus 7, 8.

Most studies of B cells have used murine B cells and focused on IgG, the most abundant antibody class in circulation (10 000 more abundant than IgE) 4. CSR to IgG has been shown to occur in secondary lymphoid tissue where antigen‐activated T helper (Th) cells interact with cognate B cells to stimulate proliferation of the B cells and then form ‘GCs’ 9, 10, 11. B‐cell proliferation is engendered by the cognate interaction of activated T cells (of one of two types, Th1 or Th2) with antigen‐specific B cells. The T cells provide a contact signal, CD40 ligand that binds to CD40 on the B cells, and secrete growth factors (cytokines) that engender B‐cell proliferation 9, 11, 12. The IgG1^+^ GC B cells undergo somatic hypermutation (SHM) and selection (affinity maturation) to secrete high‐affinity antibodies 10, 11, 13, 14.

Recent studies in the mouse have overcome the difficulty of detecting IgE^+^ cells *in vivo* using novel ultrasensitive tracking methods, revealing dramatic differences between the development of IgG^+^ and IgE^+^ cells 15, 16, 17, 18. The relevance of the conclusions from these studies to the understanding of allergic disease in humans nevertheless requires validation 3. However, CSR to IgE occurs at a very low frequency compared to IgG 4, hence the difficulty in following the subsequent development of the human IgE^+^ cells 3.

Tonsils provide a readily accessible and abundant source of human B cells from secondary lymphoid tissue. They contain a heterogeneous mixture of B cells comprising several different phenotypes, including naïve, eGC, GC, and memory B cells and plasmablasts 19. These cells can be induced to undergo CSR to IgE by stimulating with IL‐4 and anti‐CD40 *ex vivo*, mimicking the conditions required for CSR to IgE *in vivo*. The IL‐4 receptor (IL‐4R) and CD40 signaling upregulate the expression of ε germline gene transcripts (εGLTs) and mRNA for the enzyme activation‐induced cytidine deaminase (AID), two factors required for CSR to IgE 20, 21, 22, 23.

Here, we observed a wide variation in the capacity of cultured tonsil B cells, from different donors, to undergo CSR to IgE *ex vivo*. We hypothesized that this variability could be due to the variations in the phenotypic composition of stimulated tonsil B cells at the outset of the culture. Indeed, here we report a positive correlation between the proportion of eGC B cells in the purified tonsil B cells at the outset of the culture and the yields of IgE^+^ cells at the end of the cultures. We then compared the capacity of various B‐cell subsets to undergo CSR to IgE and found that B cells from different stages of development can be induced to undergo CSR to IgE. However, the intrinsic properties, namely their enhanced proliferation and IL‐4R and CD40 signaling, of GC‐derived B cells make them much more efficient in CSR to IgE.

## Methods

### Isolation of human tonsil B cells

Following informed written consent, with ethical approval from St Thomas and Guy's Research Ethics Committee, we obtained human tonsils from child donors undergoing routine tonsillectomies at the Evelina Children's Hospital, St Thomas’ Hospital, United Kingdom. We obtained verbal information on the allergic status of the donor from patients or carers at the time of consent. Mononuclear cells were separated by density centrifugation on a Ficoll gradient (GE Healthcare, Buckinghamshire, UK) and B cells isolated using 2‐aminoethylisothiouronium bromide‐treated sheep RBCs (TCS Biosciences Ltd, Buckingham, UK). B cells were >95% CD19^+^ as determined by flow cytometry (FACS).

### FACS analysis

Surface and intracellular staining of cells was performed as previously described 24. For intracellular staining, cells were fixed with 2% paraformaldehyde (Electron Microscopy Sciences, Hatfield, USA), washed, and resuspended in permeabilization buffer (PBS, 0.5% Triton X‐100, and 0.5% saponin) (Sigma‐Aldrich Ltd, Dorset, UK) for 20 min at RT. The cells were then washed with PBS + 5% goat serum and stained with fluorescently labeled antibodies (see Table S1 for the list of antibodies used) in the dark for 30 min. To determine the cell viability of the cultured cells, we used the live/dead fixable dead stain kit (Life Technologies Ltd, Paisley, United Kingdom). To measure the levels of phosphorylated STAT6 and NFκB proteins in naïve, memory, eGC, and GC B cells 24 h after culture with IL‐4 and anti‐CD40, cells were fixed, permeabilized, and stained with rabbit anti‐human p‐STAT‐6 (Tyr641) and rabbit anti‐human p‐NFκB p65 (Ser536) followed by goat anti‐rabbit IgG F(ab’)_2_ APC. Data were collected on a BD FACSCalibur or BD FACSCanto^™^ (BD Biosciences, San Jose, CA, USA) and analyzed using flowjo software version 7.6.3 (Tree Star Inc., Ashland, OR, USA).

### Cell cultures

To induce CSR to IgE, B cells were cultured as previously described 24. Briefly, freshly isolated tonsil B cells were cultured at 0.5 × 10^6^/ml in RPMI 1640 with penicillin (100 IU/ml), streptomycin (100 μg/ml), glutamine (2 mM; Invitrogen), 10% FCS (Hyclone; Hyclone Laboratories, Inc. South Logan, UT, USA), transferrin (35 μg/ml), and insulin (5 μg/ml) (Sigma‐Aldrich). B cells were stimulated with IL‐4 (200 IU/ml; R&D Europe Systems Ltd, Abingdon, UK) and anti‐CD40 antibody (0.5 μg/ml; G28.5; American Type Culture Collection, Manassas, VA, USA) for up to 12 days.

### Cell sorting

Tonsil B cells were surface‐stained with anti‐human CD27 and anti‐human CD38 on ice for 15–30 min and then naïve (CD27^−^CD38^−^), eGC (CD27^−^CD38^+^), GC (CD27^+^CD38^+/++^), and memory (CD27^+^CD38^−^) B cells were isolated using a BD FACSAria 85 micron nozzle (BD Bioscience). Sorted cells were checked for purity, counted, and cultured with IL‐4 and anti‐CD40 antibody (as above).

### Cell proliferation

Single‐cell suspensions were labeled with 10 μM carboxyfluorescein diacetate succinimidyl ester (CFSE) according to the manufacturer's instructions (CellTrace^™^ CFSE Cell Proliferation Kit; Life Technologies). After 12 days of incubation with IL‐4 and anti‐CD40, cells were harvested and acquired on a BD FACSCalibur (BD Biosciences).

### ELISA

Secreted IgE and IgG in the supernatant of CSR cultures were measured by ELISA as described previously 24, except that 3,3′,5,5′‐tetramethylbenzidine (TMB) substrate was used to develop the color reaction (R&D Europe Systems Ltd) and plates were read at 450 nm.

### RNA isolation and quantitative RT‐PCR

Total RNA was isolated from cells using the Qiagen RNeasy Mini Kit with on‐column DNase digestion (Qiagen Ltd, Manchester, UK) and reverse‐transcribed with Maxima^®^ Reverse Transcriptase enzyme (Fermentas GmbH, Hanover, Germany). RT‐PCR was performed using TaqMan MGB gene expression assays and TaqMan Universal PCR Master Mix on a 7900HT real‐time PCR machine (Applied Biosystems, Foster City, CA, USA). Gene expression was normalized to an endogenous reference gene 18S rRNA (Hs99999901_s1; Applied Biosystems). AID‐specific primers and probe were designed using the online Universal Probe Library Assay Design Centre (Roche Applied Science, Penzberg, Germany). The primer/probe sets for εGLT and γGLT were designed in‐house, and the FAM‐labeled MGB probes were purchased from Applied Biosystems and corresponding forward and reverse primers synthesized from Sigma‐Aldrich Ltd. See Table S2 for information about the primer/probe sets. SDS software was used to determine relative quantification of the target cDNA according to the 2^−(ΔΔct)^ method.

### Detection of Iε‐Cμ and Iε‐Cγ switch circle transcripts (SCTs)

CSR to IgE can be detected by the presence of SCTs, spanning from *I*ε to *IgHM* (Iε*‐*Cμ; SCT for direct switching from IgM) or to *IgHG* (Iε*‐*Cγ; SCT for sequential switching through an IgG intermediate) 25, 26, 27. Starting from the total RNA, SCT cDNAs were amplified in a 20 μl nested PCR using Phusion Flash High‐Fidelity PCR Master Mix (Thermo Scientific, Waltham, MA, USA), 1 μl cDNA, and 250 nM of forward or reverse primers (see Table S3 for information about the primer sets; round 1: Iε Fw1 and Cμ Rev1 or Iε‐Cμ SCT and Cγ Rev1; round 2: Iε Fw2 and Cμ Rev2 or Cγ Rev2). Round 1 PCR were performed at 98°C for 10 s, followed by 30 cycles of 98°C for 1 s, 68°C (Iε‐Cγ) or 65°C (Iε‐Cμ) for 10 s and 72°C for 15 s, and 1 cycle at 72°C for 1 min. PCR2 was carried out at 98°C for 10 s, followed by 20 cycles of 98°C for 1 s, 68°C (Iε‐Cγ) or 65°C (Iε‐Cμ) for 5 s and 72°C for 15 s, and 1 cycle at 72°C for 1 min. PCRs were standardized using equal amounts of RNA for the cDNA synthesis. GAPDH was amplified to check the integrity of cDNA. See Table S3 for information about the primer/probe sets used.

### Statistical analysis

Statistical analysis was performed using the one‐way anova, with Bonferroni correction, or unless otherwise stated. A *P* value of <0.05 was considered significant (*P < 0.05, **P < 0.01, ***P < 0.001).

## Results

### Induction of CSR to IgE is highly variable in tonsil B‐cell cultures

To induce CSR to IgE, we cultured freshly isolated human tonsil B cells with IL‐4 and anti‐CD40 antibody. We confirmed that our intracellular IgE staining correctly identifies IgE^+^ cells in our cultures using an antibody against the long form of membrane IgE (mIgE_L_), which recognizes an epitope on the extramembrane proximal domain (EMPD) of mIgE_L_ that is absent from secreted IgE 28 (Fig. S1).

**Figure 1:**
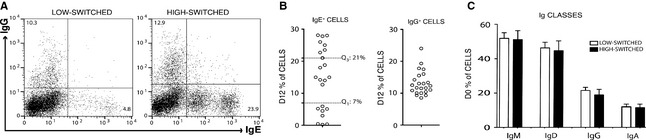
Induction of CSR to IgE is highly variable in tonsil B‐cell cultures. (A) Intracellular staining of IgE and IgG on day 12 of a total B‐cell culture that yielded low levels (low‐switched) and high levels of IgE^+^ (high‐switched) B cells. (B) IgE^+^ and IgG^+^ cell percentages on day 12 of culture. The dotted line on the IgE^+^ cell graph indicates the first quartile (Q1 < 7%; low‐switched) and the third quartile (Q3 > 21%; high‐switched). (C) FACS was used to determine the starting frequencies of each Ig class in tonsil B cells prior to culture. Data represent the mean of percentages ± SD of low‐switched (*n* = 8) and high‐switched (*n* = 8) B‐cell cultures.

We found that the capacity of tonsil B cells to undergo CSR to IgE in response to IL‐4 and anti‐CD40 is donor dependent but independent of their reported allergic status. The variation in CSR to IgE is exemplified by the FACS plots from two typical tonsil B‐cell cultures, one yielding a ‘low’ and the other a ‘high’ percentage of IgE^+^ B cells by day 12 of the culture (Fig. 1A). The yields of IgE^+^ B cells were more variable between tonsils than those of IgG^+^ B cells (Fig. 1B). To gain an understanding of this variability, we characterized the B‐cell preparations giving rise to IgE^+^ cells that fell within the first quartile (low‐switched) and the third quartile (high‐switched) of the distribution (Fig. 1B). At the outset of cell cultures, we examined the percentages of B cells expressing all five Ig classes and found there was no variability, between the low‐switched and high‐switched cultures, in the number of B cells expressing any of the Ig classes (Fig. 1C). IgE^+^ cells were not detectable.

### The frequency of eGC and memory B cells predicts the capacity of tonsil B cells to undergo CSR to IgE

**Figure 2:**
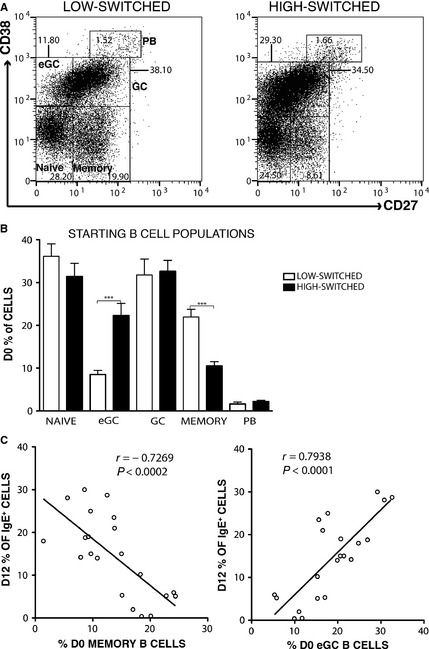
The starting frequency of eGC and memory B cells is predictive of CSR to IgE. Surface staining of CD27 and CD38 on purified tonsil B cells was used to assess the proportions of naïve, eGC, GC, and memory B cells and plasmablasts at the outset of culture. Representative FACS plots from low‐ and high‐switched cultures are shown in (A), and the data showing the mean (±SD) from low‐switched (*n* = 8) and high‐switched (*n* = 7) cell cultures are summarized in (B). (C) Correlation of day 12 IgE^+^ cell percentages with day 0 memory and eGC B‐cell percentages. Correlation analysis was performed using Spearman's rank correlation coefficient (***P < 0.001).

B cells in human lymphoid tissue are known to be heterogeneous in composition 19. Thus, we examined whether the starting proportions of naïve, eGC, GC, and memory B cells and plasmablasts (PBs) might correlate with the yield of IgE^+^ B cells at the end of culture. Figure 2A and B demonstrates that the frequency of eGC B cells was significantly greater (*P* < 0.0001) and the frequency of memory B cells significantly lower (*P* < 0.0001) in the high‐switched compared to the low‐switched cultures. The starting frequencies of naïve and GC B cells and PBs were similar between these cultures. Accordingly, the percentage of IgE^+^ cells on day 12 of the culture was positively correlated with the percentage of eGC B cells (r = 0.79, *P* < 0.0001) and negatively correlated with the percentage of memory B cells at the start of the culture (r = −0.73, *P* < 0.0002) (Fig. 2C).

### eGC and GC B cells exhibit the poorest survival but yield the highest percentage of IgE^+^ cells

**Figure 3:**
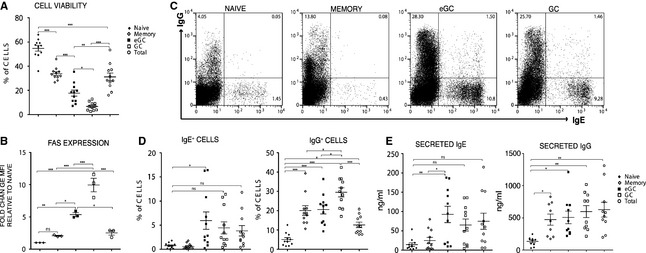
eGC and GC B‐cell cultures yield the highest percentage of IgE^+^ cells. (A) After 7 days in culture with IL‐4 and anti‐CD40, we determined, using a live/dead fixable dead stain kit in combination with the side and forward side scatter gating, the cell viability of the B‐cell cultures. (B) Fold change of MFI of anti‐FAS‐stained cells relative to naïve B cells. (C) FACS dot plots showing the intracellular staining of IgE^+^ and IgG^+^ cells after 7 days of CSR culture. (D) Percentage of IgE^+^ and IgG^+^ cells as determined by FACS analysis of the intracellularly stained day 7 naïve, memory, eGC, GC, and total B‐cell cultures. (E) IgE secretion and IgG secretion on day 7 of the cultures were analyzed by ELISA. Data represent the mean ± standard error of mean (SEM) and show the amounts of secreted IgE and IgG [ng/ml] (*P < 0.05, **P < 0.01, ***P < 0.001).

We next compared the capacity of naïve, eGC, GC, and memory B cells to undergo CSR to IgE. The four B‐cell subsets were purified by FACS and cultured, alongside unfractionated total tonsil B cells, with IL‐4 and anti‐CD40 (Fig. S2). We observed that the cell viability was relatively poor in the GC and eGC cultures, compared to the memory and naïve B‐cell cultures, with the unfractionated cells resulting in the expected intermediate yields (Fig. 3A). The increased cell death in eGC and GC cells was directly proportional to the expression levels of FAS (CD95) (Fig. 3B), a marker associated with cell death 29.

Despite the relatively poor survival, the percentage of IgE^+^ switched cells and the amounts of secreted IgE were comparable between eGC and GC B‐cell cultures but significantly greater for the eGC compared to the naïve or the memory B‐cell cultures (Fig. 3C–E). In the case of the GC cells, this difference did not reach significance, which may be attributed to the higher rate of cell death compared to the eGC cells (Fig. 3D and E). In contrast, the yields of IgG^+^ B cells and secreted IgG were significantly higher in eGC, GC and, notably, memory B cells compared to naïve B‐cell cultures (Fig. 3C–E), reflecting the high percentages of these cells at the outset of the cell culture (Fig. S3).

### Increased CSR to IgE is associated with increased cell proliferation

**Figure 4:**
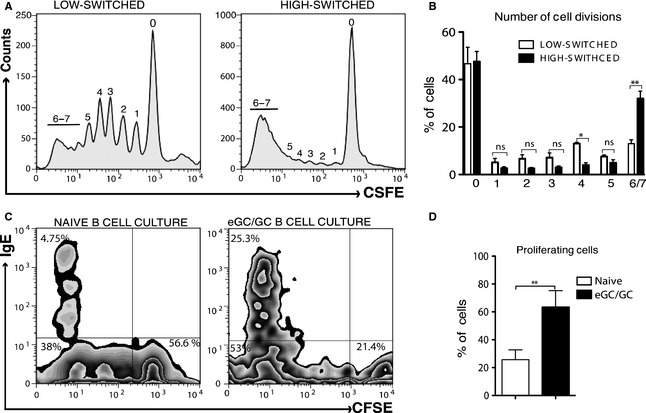
The yields of IgE^+^ cells in IL‐4 and anti‐CD40 cultures are associated with the proliferative capacity of the cultured cells. (A) Division of cells from a representative low‐switched and high‐switched tonsil B‐cell culture as determined by the CFSE dilution, whereby each peak, as indicated by the numbers, represents successive cell divisions. Data are derived from the results on day 12 of cell culture and are representative of three experiments. (B) Percentage of cells at each divisional peak in the low‐ and high‐switched tonsil B‐cell cultures. Data are derived from the results on day 12 of cell culture and represent the mean of percentages ± SD of low‐switched (*n* = 3) and high‐switched (*n* = 3) B‐cell cultures. (C) FACS plots show the proliferative capacity of the sorted naïve and total GC (eGC and GC) B cells, as determined by the CFSE dilution, and the percentage of IgE^+^ cells, as determined by intracellular staining, on day 12 of the IL‐4 and anti‐CD40 cultures. (D) The summarized percentage of proliferating cells in the naïve and eGC/GC B‐cell cultures. Data are derived from the results on day 12 of cell culture and represent the mean of percentages ± SD of naïve (*n* = 4) and eGC/GC (*n* = 4) B‐cell cultures. Statistical analysis was performed using an unpaired two‐tailed t‐test (*P < 0.05, **P < 0.01).

CSR to IgE in IL‐4‐ and anti‐CD40‐stimulated murine B cells *ex vivo* depends on the number of cell divisions, with a greater number of divisions required for IgE than for IgG 30, 31. Therefore, we examined the proliferative capacity of the high‐switched and low‐switched tonsil B cells, stained by CSFE before culture, after 12 days of stimulation with IL‐4 and anti‐CD40. We observed that high‐switched B‐cell cultures underwent robust proliferation, peaking at 6–7 cell divisions, whereas the majority of the proliferating cells from the low‐switched B‐cell culture had not undergone the same number of divisions (Fig. 4A and B).

To pursue this further, we examined the proliferative capacity of the FACS sorted naïve B cells, representing the least productive tonsil B‐cell fraction, and a mixture of eGC and GC cells representing the more productive B‐cell fractions (Figs S4 and 4B). After 12 days of culture with IL‐4 and anti‐CD40, the IgE and CFSE FACS profiles indicated that, unlike naïve B‐cell cultures, the GC‐derived B‐cell cultures had proliferated extensively and yielded a much higher percentage of IgE^+^ cells (Fig. 4C and D).

### eGC and GC B cells exhibit enhanced capacity for IL‐4 and anti‐CD40 signaling

**Figure 5:**
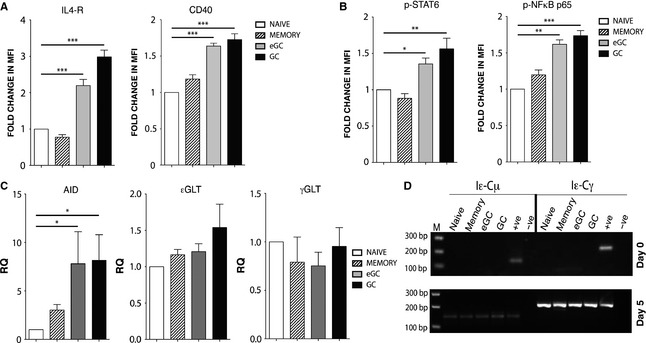
Enhanced IL‐4R and CD40 signaling in eGC and GC B cells. (A) IL‐4R and CD40 expression levels on tonsil B cells prior to culture with IL‐4 and anti‐CD40. Data show the percentage change in MFI of anti‐IL‐4R‐ and anti‐CD40‐stained cells relative to naïve B cells. (B) STAT6 and NFκB phosphorylation levels 24 h poststimulation with IL‐4 and anti‐CD40. Data show the fold change in MFI of anti‐p‐STAT6 (Tyr641)‐ and anti‐p‐NFκB p65 (Ser536)‐stained cells relative to naïve B cells. They represent the mean ± SD and are derived from three different tonsils. (C) RT‐PCR analysis of AID, εGLT, and γGLT expression 24 h poststimulation with IL‐4 and anti‐CD40. Data represent the mean ± SD of the relative quantification (RQ) and are derived from three different tonsils. (D) RNA was isolated from the sorted naïve, memory, eGC, and GC B cells on day 0 (preculture) and day 5 of cell culture with IL‐4 and anti‐CD40 antibody, The presence of Iε‐Cμ SCTs (167 bp; direct switching) and Iε‐Cγ SCTs (202 bp; sequential switching) was determined by nested PCR. Because of the nature of the nested PCR, the intensity of the PCR band does not necessarily reflect the amounts of switching occurring in our cultures. The positive control was cDNA from an IL‐4‐ and anti‐CD40‐stimulated tonsil B‐cell culture that yielded high percentages of IgE^+^ cells. The negative control was distilled H_2_O. Data are representative of three experiments. M = size marker, and bp = the size of the cDNA in base pairs (*P < 0.05, **P < 0.01, ***P < 0.001).

Given the important role of IL‐4 and anti‐CD40 in the stimulation of CSR to IgE, we examined the capacity of the different tonsil B‐cell fractions to respond to these signals. Prior to culture with IL‐4 and anti‐CD40, the expression of the IL‐4 receptor (IL‐4R) and CD40 in eGC and GC B cells was significantly higher than in naïve and memory B cells (Fig. 5A). We also analyzed the phosphorylation of STAT6 and NFκB, two important downstream signal transducers of IL‐4R and CD40, respectively 22, 23, 32, 33, by FACS. Figure 5B demonstrates that the activity of these proteins reflects the relative levels of expression of IL‐4R and CD40.

To assess the outcome of these signaling pathways, we used RT‐PCR to measure the relative levels of expression of εGLT 22, 34, 35 and AID 21. We also assayed γGLT to see whether this differed between the B‐cell fractions. The expression levels of the AID mRNA, but not εGLT or γGLT, were significantly upregulated in the GC and eGC cells compared to the naïve and memory B cells (Fig. 5C).

To demonstrate unequivocally that IL‐4 and anti‐CD40 stimulates *de novo* CSR to IgE *in vitro*, we assayed switch circle transcripts (SCTs), transient by‐products of CSR 25. We assayed Iε‐Cμ and Iε‐Cγ PCR in naïve, memory, eGC, and GC sorted B cells on days 0, 1, and 5 of the culture with IL‐4 and anti‐CD40 using a nested PCR. Neither Iε‐Cμ nor Iε‐Cγ transcripts could be detected in any of the fractions on day 0 or day 1 (not shown), but they both appeared in all four fractions by day 5 (Fig. 5D). These results demonstrate that IL‐4 and anti‐CD40 stimulate both *de novo* direct (IgM→IgE) and sequential (IgM→IgG→IgE) CSR to IgE in the cultured tonsil B cells.

## Discussion

IL‐4 and anti‐CD40 culture of tonsil B cells mimics the conditions for the induction of CSR to IgE *in vivo* 5, 36. In this study, we observed a wide variation in the yields of IgE^+^ B cells in IL‐4 and anti‐CD40 cultures of tonsil B cells from different donors. The yields of IgE^+^ cells at day 12 of the cultures were negatively correlated with the proportion of memory B cells and positively correlated with the proportion of eGC B cells at the outset of the cell culture. This can be rationalized by the intrinsic properties of these cells, namely the relative strength of the response to IL‐4 and anti‐CD40 signaling and the differing rates of cell proliferation.

Prior to culture with IL‐4 and anti‐CD40, eGC and GC B cells already exhibit upregulated IL‐4R and CD40 expression. These expression levels, upon stimulation, were reflected on their activation of critical signal transduction factors, STAT6 and NFκB, and AID mRNA, which together are required for CSR to IgE, giving eGC and GC B cells a ‘head start’ over the other B‐cell fractions. STAT6 and NFκB bind directly to the ε germline gene to activate transcription 22, 34, 35. The resulting transcription is involved in remodeling the chromatin in the region of the gene to make it accessible for subsequent recombination, and the transcript itself participates in the mechanism of CSR 37.

CSR to IgE depends on the number of cell divisions and requires a greater number for IgE compared to IgG 30, 31, suggesting that another important factor contributing to the observed differences in the yields of IgE^+^ cells is the proliferative capacity of the cultured cells. Our data clearly demonstrate that tonsil B‐cell cultures that yield high percentages of IgE^+^ cells and specifically cells from the GC compartments undergo robust proliferation. We cannot exclude the possibility of an antigen driving the proliferation of the switching B cells in our high‐switched cultures and contributing to the higher yields of IgE^+^ cells. However, it is unlikely to occur via BCR cross‐linking *ex vivo*, as this inhibits CSR to IgE 38, 39.

It is well documented that B cells within GCs proliferate very robustly and undergo SHM and recombination specifically (with rare exceptions) in Ig genes, a mechanism that has evolved to diversify the antibody repertoire prior to affinity maturation in the immune response 10, 14, 40. These processes are known to predispose B cells to apoptosis, indicated here by the high rates of cell death in eGC B‐cell cultures and even higher rates of cell death in GC B‐cell cultures. Activated B cells are known to express Fas ligand 41, and the interaction of Fas with its ligand has been implicated during the negative selection of B cells in the GCs 42, 43. The differential expression of Fas by eGC and GC B cells, although it may not be a specific mechanism that fully accounts for cell death in B cells from the GC compartments, may explain the differing rates of cells death in these cultures. Therefore, considering that eGC and GC B‐cell cultures yield comparable percentages of IgE^+^ cells, the differences in the cell viability of these B cells might explain why a tonsil B‐cell culture starting with a higher proportion of eGC B cells would yield more IgE^+^ B cells.

We were unable to detect IgE^+^ cells in tonsil B cell at the outset of the cultures, and we only see IgE^+^ cells after 3–4 days in IL‐4 and anti‐CD40 cultures, approximately the time when we can also detect ε circle transcripts and other markers of CSR to IgE (P. Hobson and D. Fear, unpublished). SCT analysis demonstrated *de novo* CSR from IgG as well as from IgM in our B‐cell cultures. The IgG^+^ B‐cell precursors may have been ones that were already present at the outset of the cultures, or ones that resulted by *de novo* switching from IgM to IgG en route to IgE *ex vivo*. Although there may be a higher frequency of CSR to IgG than to IgE 44, 45, we observed that the mean yield of IgG cells is similar to that of IgE. This probably reflects the onward switching from IgG to IgE. The dynamic interplay between SHM and CSR from the different isotypes to IgE is a problem that can be tackled in future studies with this *ex vivo* system.

After CSR, the B cells must undergo plasma cell differentiation to secrete IgE. This evidently occurs in our cultures, as indicated by the secretion of IgE. Indeed, in our FACS profiles we observe two IgE^+^ cell populations (Fig. 1A), which are reminiscent of the two IgE^+^ cell populations observed in mice 18, and we have characterized these cells as plasma cell precursors in our ongoing work.

Understanding the mechanisms of CSR to IgE is essential for a full understanding of the pathophysiology of allergic disease. Our findings open up new avenues of research on human B cells to validate the conclusions from mouse models of this disease or discover inherent differences between the two species to account for the higher risk of developing allergic disease in humans 3.

## Author contributions

F.R. designed and performed the experiments, analyzed the data, and wrote the manuscript. N.N.U. and P.S.H. performed experiments and analyzed the data. Y.C., D.M, H.B., and CK performed experiments. B.J.S. and D.J.F provided guidance and critically reviewed the manuscript, and H.J.G. designed the experiments, analyzed the data, and wrote the manuscript. All authors reviewed the final manuscript.

## Conflicts of interest

The authors declare that they have no conflicts of interest.

## Supporting information


**Figure S1.** Identification of IgE^+^ B cells by intracellular FACS staining.
**Figure S2.** FACS sorting of naïve, memory, eGC and GC B cells cultures.
**Figure S3.** Day 0 frequency of IgM^+^, IgG^+^, IgA^+^ expressing B cells within the naïve, memory, eGC and GC tonsil B cells.
**Figure S4.** FACS sorting of naive and eGc/GC B cells for proliferation cultures.
**Table S1.** List of antibodies used in flow cytometry experiments.
**Table S2.** List of primers and probes used for qRT‐PCR.
**Table S3.** List of primers and probes used for the detection of SCTs.
