# Supplementary material for: Intrinsic properties of germinal center‐derived B cells promote their enhanced class switching to IgE
Source: Allergy. 2015 Jul 24;70(10):1269–77. doi: 10.1111/all.12679 (PMC4744720; doi:10.1111/all.12679)
Supplement: Supplementary file 1 — Figure S1. Identification of IgE+ B cells by intracellular FACS staining. Figure S2. FACS sorting of naïve, memory, eGC and GC B cells cultures. Figure S3. Day 0 frequency of IgM+, IgG+, IgA+ expressing B cells within the naïve, memory, eGC and GC tonsil B cells. Figure S4. FACS sorting of naive and eGc/GC B cells for proliferation cultures. Table S1. List of antibodies used in flow cytometry experiments. Table S2. List of primers and probes used for qRT‐PCR. Table S3. List of primers and probes used for the detection of SCTs. [file ALL-70-1269-s001.doc]

**Supplemental Materials**

**Ramadani et al. 2015**

**Supplementary Tables**

**Table S1:** List of antibodies used in flow cytometry experiments.

| **Antibodies** | **Conjugate** | **Supplier** | **Clone** | **Cat No.** |
| --- | --- | --- | --- | --- |
| anti-CD19 | Alexa Fluor 647 | Biolegend | HIB19 | 302222 |
| anti-CD20 | FITC | BD Pharmingen | 2H7 | 555622 |
| anti-CD27 | FITC | Dako | Polyclonal | M-T271 |
| anti-IgA | APC | Miltenyi Biotec | Polyclonal | 130-093-113 |
| anti-IgG | APC | Miltenyi Biotec | Polyclonal | 130-093-194 |
| anti-IgE | FITC | Vector Laboratories | Polyclonal | FI-3040 |
| anti-IgE | Unconjugated | Vector Laboratories | Polyclonal | AI-3040 |
| anti-IgE | BIOTIN | Vector Laboratories | Polyclonal | BA-3040 |
| anti-mIgE_L_ | BIOTIN | [**^1^**](#_ENREF_1) | 4B12 |  |
| anti-IgD | RPE | Serotec | Polyclonal | 203009 |
| anti-IgM | FITC | Dako | Polyclonal | F0058 |
| anti-IgM | APC | Biolegend | MHM88 | 314510 |
| anti-CD38 | PE | Biolegend | HIT3 | 303506 |
| anti-CD40 | APC | BioLegend | HB14 | 313007 |
| anti-IL-4Rα | APC | R&D | Polyclonal | FAB2304 |
| p-STAT6 ( Tyr641) Rabbit mAb | Unconjugated | Cell Signalling |  | 9361 |
| p-NF-κB p65 (Ser536) Rabbit mAb | Alexa Fluor 647 | Cell Signalling | 93H1 | 4887 |
| Goat anti-Rabbit IgG F(ab’)2 | APC | Santa Cruz |  | Sc-3846 |

**Table S2:** List of primers and probes used for qRT-PCR.

| **Gene** | **FORWARD primerS** | **RevERSE primerS** | **Probe** |
| --- | --- | --- | --- |
| **AID** | 5’-GGACTTTGGTTAT  C TTCGCAAT-3’ | 5’-GTCGGGCACAGTC  GTAGC-3’ | **UPL** 1 |
| **εGLT** | 5′-CTGTCCAGGAAC  CCGACAGA-3′ | 5′-TGCAGCAGCGGG  TCAAG-3′ | 5′-AGGCACCAAA  TG-3′ |
| **γGLT** | 5’-CCAGGGCAGGGT  CAGCA-3’ | 5’- CGATGGGCCCTT  GGTGGA -3’ | 5′-CTCAGCCAGGAC  CAAG-3′ |

**Table S3:** List of primers and probes used for the detection of SCTs

| **PRIMER NAME** | **PRIMER SEQUENCES** |
| --- | --- |
| IεF1 | 5’-CCACGGTTACTGATCATCTGGGAGC-3’ |
| IεF2 | 5’-CTGATCATCTGGGAGCTGTCC-3’ |
| CμR1 | 5’-CCACGCTGCTCGTATCCGAC-3’ |
| CμR2 | 5’-GGGGAATTCTCACAGGAGAC-3’ |
| CγR1 | 5’-CGTTGCTGAGGGAGTAGAGTCC-3’ |
| CγR2 | 5’-CACCGTCACCGGTTCGGGG-3’ |

F= Forward; R=Reverse

**Supplementary Figures**

**
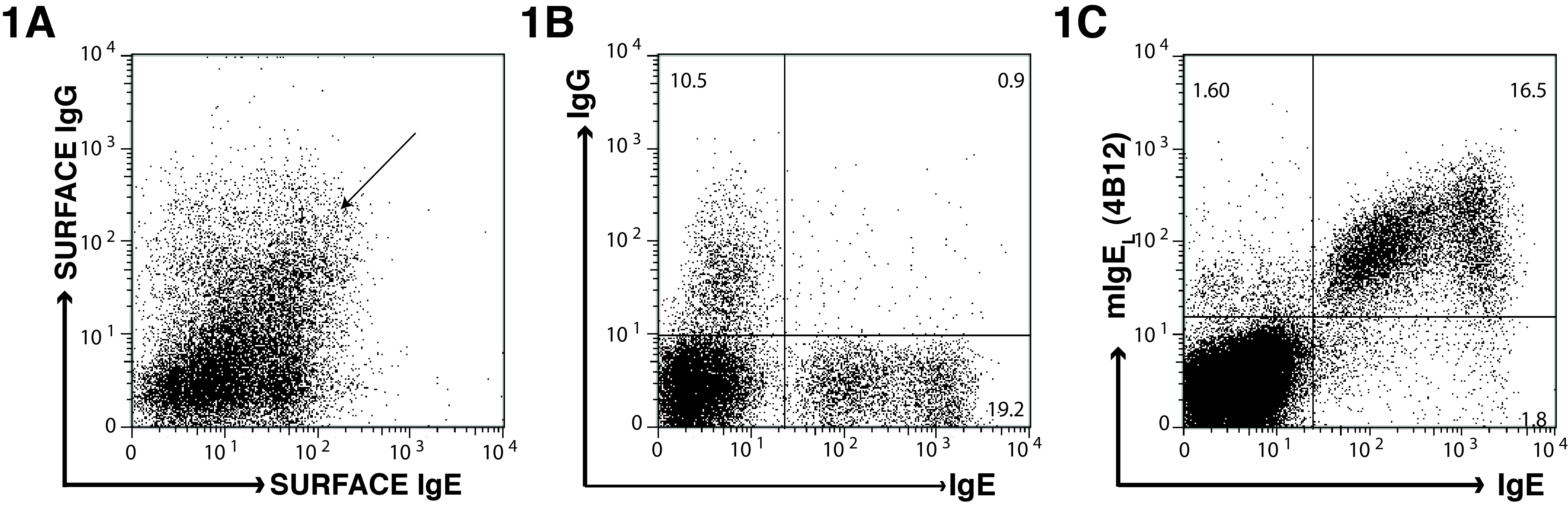
**

**Figure S1.**  **Identification of IgE^+^ B cells by intracellular FACS staining**

**(A)** On day 12 of culture with IL-4 and anti-CD40, cells were harvested, washed twice with PBS + 5% goat serum and surface stained with anti-IgG and a polyclonal anti-IgE antibody. Arrows indicate the cytophilic IgE, secreted IgE bound to membrane CD23 on IgG^+^ B cells, making it difficult to correctly identify IgE-expressing cells. **(B)** Day 12 tonsil B cell cultures were harvested, washed with PBS + 5% goat serum twice, fixed/permeabilised and stained intracelluarly for IgE and IgG. **(C)** To confirm that our intracellular staining method correctly identifies the IgE^+^ cells we surface stained the day 12 cultured B cells with anti-mIgE_L_ (4B12) antibody, which bind the long form of membrane IgE (mIgE_L_), followed by intracellular staining with polyclonal anti-IgE. Data are representative of 5 different experiments.

**
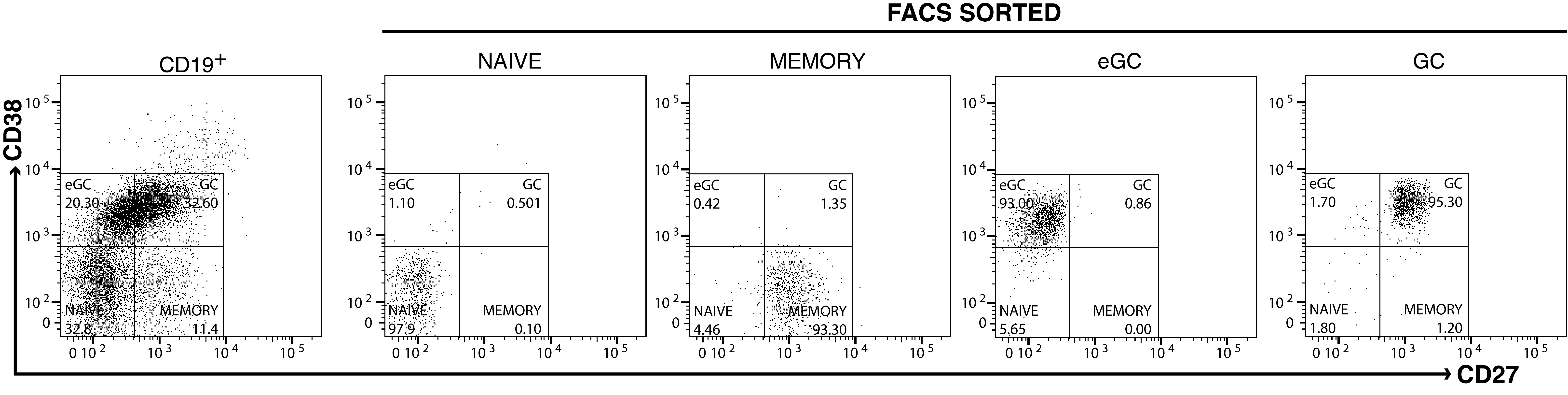
**

**Figure S2.** **FACS sorting of naïve, memory, eGC and GC B cells cultures.** CD19^+^ cells were FACS sorted based on their CD27 and CD38 surface expression into naïve (CD19^+^CD27^-^CD38^-^), eGC (CD19^+^CD27^-^CD38^+^), GC (CD19^+^CD27^+^CD38^+/++^) and memory B cells (CD19^+^CD27^+^CD38^-^). The data shows the purity of sorted cells as measured on BD FACSAria. The sorted cells were then counted and cultured cultured for up to 7 days with IL-4 and anti-CD40. Data are representative of 5 different experiments.


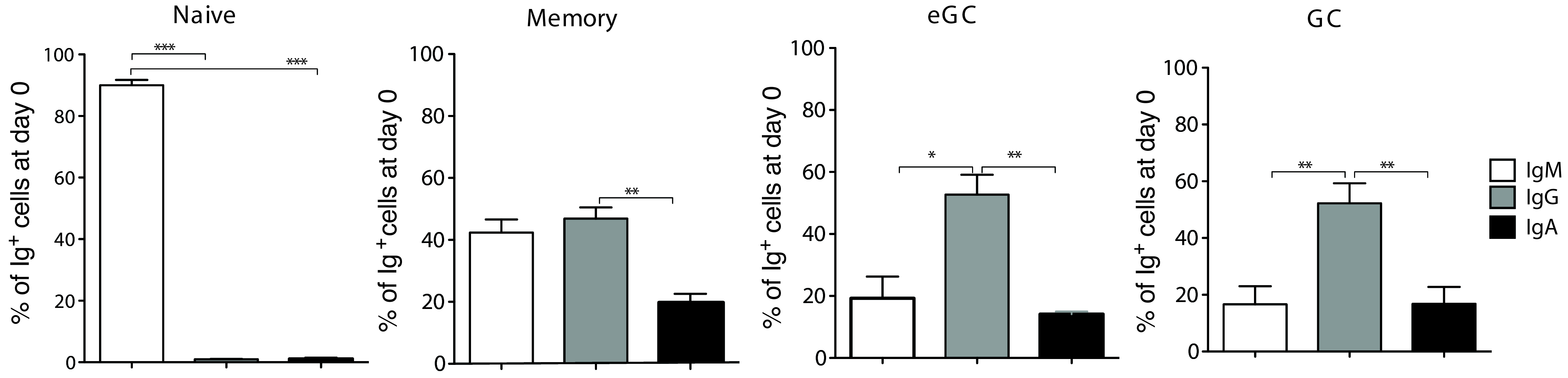


**Fig. S3.** **Day 0** **frequency of IgM^+^, IgG^+^, IgA^+^ expressing B cells within the naïve, memory, eGC and GC tonsil B cells**. Freshly isolated tonsil B cells were surface stained for CD27 and CD38 and either anti-IgM, anti-IgG or anti-IgA antibodies. Based on the CD27 and CD38 expression we gated the naïve, memory, eGC and GC tonsil B cells in order to determine the day 0 frequency of IgM^+^, IgG^+^, IgA^+^ expressing B cells within each of these populations . Data represent the mean +/- SD and are derived from 3 different tonsils.

**
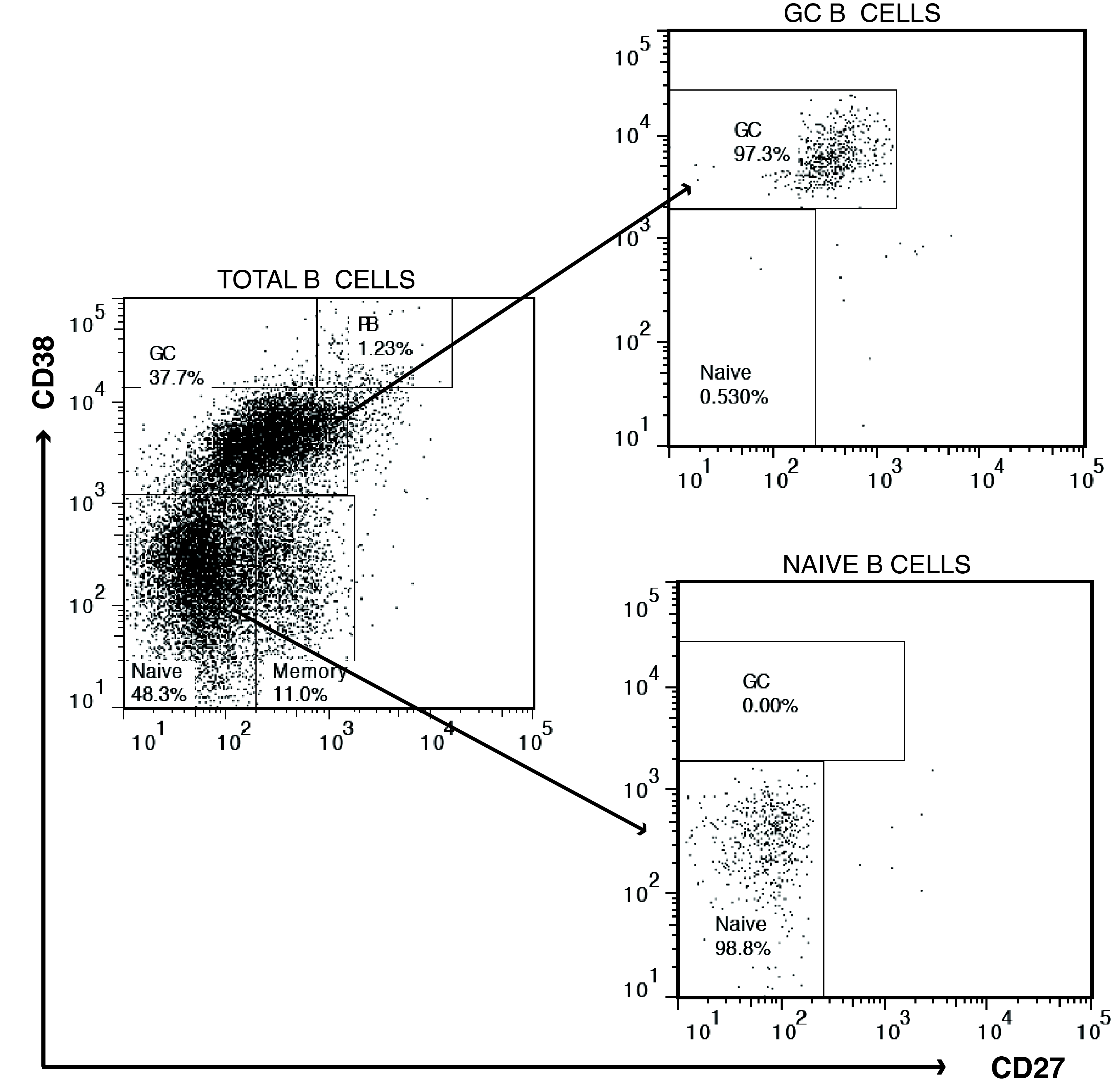
**

**Figure S4.** **FACS sorting of naive and eGc/GC B cells for proliferation cultures.** Tonsil B cells were FACS sorted, based on their CD27 and CD38 surface expression, into naïve (CD27^-^CD38^-^) and GC derived B cells (eGC and GC B cells: CD27^-/+^CD38^+/++^). The sorted naïve and GC B cells were then CFSE stained and cultured with IL-4 and anti-CD40.

**References**

1. Chen JB, Wu PC, Hung AF, et al. Unique epitopes on C epsilon mX in IgE-B cell receptors are potentially applicable for targeting IgE-committed B cells. *J Immunol*. 2010;184(4):1748-1756. Prepublished on 2010/01/20 as DOI 10.4049/jimmunol.0902437.
